# Impact of combining Lenvatinib with Transarterial chemoembolization for unresectable hepatocellular carcinoma

**DOI:** 10.12669/pjms.39.6.7944

**Published:** 2023

**Authors:** Jianwu Long, Longfei Liu, Xuefeng Yang, Xianzhou Lu, Lei Qin

**Affiliations:** 1 Jianwu Long, Department of General Surgery, The First Affiliated Hospital of Soochow University, Soochow, Jiangsu province, P.R. China; 2 Longfei Liu, Department of Hepatobiliary Surgery, The Affiliated Nanhua Hospital, Hengyang Medical School, University of South China, Hengyang, Hunan province, P.R. China; 3 Xuefeng Yang, Department of Hepatobiliary Surgery, The Affiliated Nanhua Hospital, Hengyang Medical School, University of South China, Hengyang, Hunan province, P.R. China; 4 Xianzhou Lu Department of General Surgery, Hengyang County People’s Hospital, Hengyang, Hunan province, P.R. China; 5 Lei Qin, Department of General Surgery, The First Affiliated Hospital of Soochow University, Soochow, Jiangsu province, P.R. China

**Keywords:** Transcatheter arterial chemoembolization, TACE, Lenvatinib, Unresectable hepatocellular carcinoma, HCC

## Abstract

**Objectives:**

To investigate the impact of combining lenvatinib with transarterial chemoembolization (TACE) for unresectable hepatocellular carcinoma (HCC).

**Methods:**

This was a retrospective observational study which reviewed the medical records of 103 unresectable HCC patients from January 2017 to June 2020 in The First Affiliated Hospital of Soochow University. It included 46 patients who received TACE plus lenvatinib and 57 patients who received TACE alone. The levels of serum indicators, clinical effect, adverse events, overall survival (OS), and progression-free survival (PFS) were compared between the two groups.

**Results:**

AFP and VEGF levels in the TACE+lenvatinib group post-treatment were significantly lower than the TACE group (*P*<0.05). The clinical efficacy in the TACE+lenvatinib group (69.57%) was higher than that in the TACE group (40.35%) post-treatment (*P*<0.05). There were significant differences in hypertension, diarrhea, and bleeding (gingiva) between the two groups (*P*<0.05). There were no significant differences in one or two year PFS rate or one year OS between groups (*P*>0.05), while the two years survival rate in the TACE+lenvatinib group was significantly higher than that in the TACE group (*P*<0.05).

**Conclusions:**

TACE combined with lenvatinib have a high clinical effective rate, with reduced AFP and VEGF levels, higher two year survival rate, and acceptable incidence of adverse events.

## INTRODUCTION

Hepatocellular carcinoma (HCC), the most common type of primary liver cancer, is the sixth most common cancer and the third leading cause of cancer death worldwide.^1^ The incidence of HCC has been report to be 5.1 per 100,000 person-years in Europe and 17.7 per 100,000 person-years in eastern Asia, and it has increased dramatically over the past three decades and the trend is expected to continue through 2030.^2^,^3^ Surgery is not recommended for advanced HCC by current guidelines because it may lead to greater complications and early death.^4^ Transarterial chemoembolization (TACE), as a palliative treatment for HCC, has long been recommended as a first-line treatment for patients with unresectable HCC.^5^,^6^ Studies have shown that compared with best supportive care, TACE was associated with longer survival in patients with unresectable HCC.^7^,^8^

Studies also found that TACE can promote immunogenic cell death and induce a T-cell response against tumor cells, increasing the anti-tumor activity of immune checkpoint inhibitors.^9^ However, TACE alone is not effective for some patients when regarded as systemic therpay.^10^ Lenvatinib, an oral multikinase inhibitor, has been reported to significantly improve the overall survival and median progression-free survival of the patients with HCC.^11^ Moreover, it is demonstrated to have noninferiority compared to sorafenib as a first-line treatment for unresectable HCC.^12^,^13^ Thus, lenvatinib holds promise for treating unresectable HCC. Recently, the safety and efficacy of TACE combined with lenvatinib for patients with unresectable HCC was investigated.^14^,^15^ However, data on the efficacy of TACE in combination with lenvatinib are insufficient. Therefore, we aimed to compare the efficacy of TACE plus lenvatinib versus TACE alone for patients with unresectable HCC in this study.

## METHODS

This was a retrospective observational study and the medical records of 103 unresectable HCC patients from January 2017 to June 2020 attended in The First Affiliated Hospital of Soochow University were reviewed. The patients were screened according to the inclusion and exclusion criteria, and they were divided into two groups based on the treatment received: TACE+lenvatinib group (n= 46, patients who received TACE plus lenvatinib) and TACE group (n=57, patients who received TACE alone).

### Ethical Approval:

The study was approved by the ethics committee of the hospital (No. 2022-KY-159, Date: 2022-12-27), and written informed consent was waived because of the retrospective nature of the study.

### Inclusion Criteria:

- 18<age<75.
- Patients diagnosed with unresectable HCC based on pathological or non-invasive assessment.^16^
- Liver Function scored as Child-Pugh Class-A and B.
- The Eastern Cooperative Oncology Group (ECOG) score was 0-1.
- Expected survival time ≥ three months.

### Exclusion Criteria:

- Patients with any other malignant tumors in addition to HCC;
- Patients with contraindication of TACE or lenvatinib;
- Patients with severe dysfunction of heart, kidney or other organs;
- Patients with severe mental disorders;
- Pregnant or lactating women;
- Patients with severe jaundice and massive ascites;
- Patients treated with other therapies simultaneously, such as programmed cell death 1 (PD-1) antibody, radiofrequency ablation and other immunotherapies, etc.
- Patients with incomplete medical records.

### TACE group:^15^

Patients in the TACE group received TACE alone. The surgeon with more than eight years of experience inserted a 5-French catheter percutaneously into the femoral artery by using the Seldinger technique, and then injected a contrast agent for digital subtraction angiography to determine the location, size, and blood supply of the tumor. Then the surgeon slowly injected 50 mL of diluted 5-fluorouracil hydrate and oxaliplatin through the catheter to embolize the blood vessel. When necessary, drug-loaded microspheres or gelatin sponge particles were used for arterial embolization. Antiemetics, liver protection, and stomach protection were given postoperatively. Two TACE treatments constituted a course of treatment, with an interval of four to six weeks.

### TACE+lenvatinib group:^14^

Patients in the TACE+lenvatinib group received lenvatinib (Eisai Co. Ltd.; specification: 10mg/20 capsule/box) depending on their body weight one week after TACE treatment. The dosage of lenvatinib for patients ≥60 kg and <60 kg were 12 mg and 8 mg once daily, respectively. If there were no obvious symptoms such as nausea, vomiting and fever caused by TACE, lenvatinib was discontinued for three days before each TACE treatment, and then resumed. The patients were treated continuously for six weeks as a course of treatment, and the medication was adjusted according to any adverse reactions.

### Observational Indicators:

Serum indicators: The levels of vascular endothelial growth factor (VEGF), alanine aminotransferase (ALT) and α-fetoprotein (AFP) before and after treatment were collected. Clinical effect: The clinical effect was evaluated by the Response Evaluation Criteria in Solid Tumors (RECIST).^17^

### Complete response (CR):

CT showed no target lesions.

### Partial response (PR):

The sum of lesion diameters (LD) decreased by more than 30% compared with baseline sum LD.

### Stable disease (SD):

The sum of LD decreased by less than 30% or increased by less than 20%.

### Progressive disease (PD):

Increase in sum of LD by more than 20% or appearance of new lesions. Total effective rate = (Number of CR patients + Number of PR patients) / Total number of patients × 100%.

### Adverse events:^18^

Medical records about adverse events including abdominal pain, fever, nausea and vomiting, fatigue, hypertension, hand-foot syndrome, diarrhea, bleeding (gingiva), decreased albumin, decreased PLT, decreased WBC, and elevated AST were collected.

### Overall survival (OS) and progression-free survival (PFS):

Patients were followed up for two years after treatment and medical records on OS and PFS were collected. PFS was defined as the time from the first treatment to diagnosis of disease progression or death or the end of follow-up. OS rate= Number of OS patients/Total number of patients × 100%. PFS rate = Number of PFS patients/Total number of patients × 100%.

### Statistical Analysis:

Statistical analysis was conducted by SPSS 23.0 (IBM, USA). Normally distributed and homogeneous measurement data were presented as mean ± SD, and Student’s t-test was used to compare the differences between the two groups. Non-normally distributed measurement data were presented as median (range), and Mann-Whitney U-test was used to compare the differences between the two groups. Paired-sample t-test was used to compare the differences within the same group before and after treatment. Count data were presented as frequency and percentage, and Chi-square test or Fisher’s exact test were used to compare the differences between the two groups. All statistical analysis were two-sided and *P*<0.05 was considered as statistically significant different.

## RESULTS

There were 29 males and 17 females aged 34-70 years (51.52±6.31 years) in the TACE+lenvatinib group, and there were 34 males and 23 females aged 32-68 years (52.12±5.71 years) in the TACE group. There were no statistically significant differences in baseline data between groups (*P* > 0.05; Table-I).

**Table-I:** Patient baseline data.

Variables	TACE+lenvatinib group (n=46)	TACE group (n=57)	t/	P
Gender, n (%)			-0.348	0.728
Male	29 (63.04)	34 (59.65)		
Female	17 (36.96)	23 (40.35)		
Age, years, mean ± SD	51.52±6.31	52.12±5.71	-0.507	0.614
Body weight, kg, n (%)			0.004	0.997
<60 kg	25 (54.35)	31 (54.39)		
≥60 kg	21 (45.65)	26 (45.61)		
ECOG score			-0.184	0.854
0	37 (80.43)	45 (78.95)		
1	9 (19.57)	12 (21.05)		
Child-Pugh class, n(%)			-0.220	0.826
A	41 (89.13)	50 (87.72)		
B	5 (10.87)	7 (12.28)		

### Serum indicators post-treatment:

There were no significant differences in AFP, ALT or VEGF between the two groups pre-treatment (*P*>0.05). Post-treatment, AFP, ALT and VEGF levels in each group were lower than pre-treatment (*P*<0.001). Post-treatment, AFP and VEGF levels in the TACE+lenvatinib group were significantly lower than the TACE group (*P*<0.05; Table-II).

**Table-II:** Comparison of serum indicators between groups.

Variables	TACE+lenvatinib group (n=46)	TACE group (n=57)	Z	P
***AFP (ng/mL)***				
Pre-treatment	899.76(717.17-1017.50)	891.47(765.61-990.50)	-0.229	0.819
Post-treatment	191.42(178.75-220.49)^a^	310.00(296.20-367.00)^a^	-7.712	<0.001
***ALT (U/L)***				
Pre-treatment	59.56(44.75-79.26)	60.13(47.00-75.00)	-0.027	0.979
Post-treatment	45.21(26.75-65.56)^a^	48.18(35.00-63.00)^a^	-0.808	0.419
***VEGF (ng/mL)***				
Pre-treatment	275.50(262.00-304.75)	270.56(243.50-302.00)	-0.841	0.400
Post-treatment	137.50(122.75-168.83)^a^	224.17(199.00-261.00)^a^	-8.715	<0.001

Data were presented as median (range).

a P < 0.001, compared with the same group pre-treatment.

### Clinical effect:

The clinical efficacy in the TACE+lenvatinib group (69.57%) was higher than the TACE group (40.35%) post-treatment (*P*<0.05; Table III).

**Table-III:** Comparison of clinical effect between the two groups (n,%).

Variables	TACE+lenvatinib group (n=46)	TACE group (n=57)	Fisher	P
CR	5 (10.87)	2 (3.51)		
PR	27 (58.69)	21 (36.84)		
SD	9 (19.57)	24 (42.11)		
PD	5 (10.87)	10 (17.54)		
Total effective rate	32 (69.57)	23 (40.35)	9.454	<0.05

### Adverse events post-treatment:

There were no significant differences in abdominal pain, fever, nausea and vomiting, fatigue, decreased albumin, PLT, WBC, elevated AST, and hand-foot syndrome between the two groups (*P*>0.05), while there were significant differences in hypertension, diarrhea, and gingival bleeding between the two groups (*P*<0.05; Table-IV).

**Table-IV:** Comparison of adverse events post-treatment (n, %).

Variables	TACE+lenvatinib group (n=46)	TACE group (n=57)	χ^2^/Fisher	P
Abdominal pain	19 (41.30)	23 (40.35)	0.010	1.000
Fever	12 (26.09)	15 (26.32)	0.001	1.000
Nausea and vomiting	13 (28.26)	15 (26.32)	0.049	0.828
Fatigue	24 (52.17)	21 (36.84)	2.432	0.162
Decreased albumin	22 (47.83)	27 (47.37)	0.002	1.000
Decreased PLT	16 (34.78)	19 (33.33)	0.024	1.000
Decreased WBC	13 (28.26)	12 (21.05)	0.720	0.489
Elevated AST	15 (32.61)	16 (28.07)	0.249	0.669
Hypertension	6 (13.04)	0 (0.0)	7.895	<0.05
Hand-foot syndrome	3 (6.52)	0 (0.0)	3.829	0.086
Diarrhea	5 (10.87)	0 (0.0)	6.512	<0.05
Gingival bleeding	7 (15.22)	0 (0.0)	9.306	<0.05

### OS and PFS post-treatment:

There were no significant differences in one or two year PFS rates between the two groups (*P* > 0.05). There was no significant difference in 1-year survival between the two groups (*P* > 0.05), but the two year survival rate in the TACE+lenvatinib group was significantly higher than that in the TACE group (*P*<0.05; Table-V).

**Table-V:** Comparison of survival between groups (n, %).

Variables	TACE+lenvatinib group (n=46)	TACE group (n=57)	χ^2^	P
***OS***				
1-year survival	40 (86.96)	41 (71.93)	3.422	0.090
2-year survival	34 (73.91)	29 (50.88)	5.687	0.025
***PFS***				
1-year PFS	35 (76.09)	35 (61.40)	2.521	0.139
2-year PFS	23 (50.00)	18 (31.58)	3.605	0.070

## DISCUSSION

In this study, we found that treatment with TACE plus lenvatinib had favorable outcomes as it was associated with lower AFP and VEGF levels, higher long-term survival and no additional adverse events. As the most common type of liver cancer, HCC accounts for more than 90% of primary liver cancers, which poses a serious threat to human health.^19^ Studies have shown that VEGF holds diagnostic and prognostic potential for HCC as high serum VEGF levels can predict poor prognosis of HCC patients.^20^,^21^ A recent study also reported that AFP has shown survival benefits with lenvatinib treatment in patients with hepatitis B virus-related unresectable HCC.^22^ Our results showed that AFP and VEGF levels in patients receiving TACE+lenvatinib treatment were significantly lower than the patients who received TACE alone.

However, the findings of our study were inconsistent with those of Fu et al^14^ who found no significant difference in AFP levels between the two groups. The reason for the different findings may be due to the different patient characteristics as our study had higher baseline AFP levels. Our study also showed that the clinical efficacy in the TACE+lenvatinib group was significantly higher than that in the TACE group, which was generally consistent with those of Chen et al.^15^ TACE is considered the guideline-recommended care for intermediate-stage HCC patients.^23^ TACE delivers chemotherapy drugs and embolic agents thereby blocking the blood supply of the tumor and inhibiting tumor growth.^24^ However, TACE alone cannot eliminate the residual cancer cells in the lesion, because new collateral circulation is easily established post-surgery. Lenvatinib, an angiogenesis inhibitor, inhibits VEGF and tumor fibroblast growth factor signaling pathways in unresectable HCC.^25^ The synergistic effect of the combination can inhibit tumor growth more effectively.

Abdominal pain, fever, nausea and vomiting, fatigue, decreased albumin, PLT, WBC, elevated AST, and hand-foot syndrome are common adverse events in patients receiving TACE. In our study, we found these adverse events occurred in both groups, but patients who received TACE+lenvatinib were more likely to experience hypertension, diarrhea and gingival bleeding. These adverse events may be due to lenvatinib as hypertension, diarrhea and gingival bleeding have been reported as common adverse events in unresectable HCC patients treated with lenvatinib.^26^,^27^ A recent meta-analysis showed that lenvatinib-induced hypertension may be associated with better prognosis.^28^ Lenvatinib-induced adverse events could be managed through regular monitoring, preventive measures and symptomatic management.^27^

Lenvatinib shows promise for patients with HCC as numbers of studies have shown its benefits in prolonging the OS and PFS of the patients.^11^,^29^,^30^ Similarly, both Fu et al^14^ and Chen et al^15^ found that TACE+lenvatinib was superior to TACE alone in terms of OS and PFS, whereas we found this only in terms of 2-year survival. We believe that the reasons may be because of different analytical methodologies. For Chen et al^15^, a propensity score-matched analysis was used. Furthermore, the conflicting findings may also be attributed to different treatment duration and protocols of TACE and lenvatinib administration.

### Limitations:

The small sample size of the study may limit the generalization of the research outcomes. Additionally, this was a retrospective study with limited data, which may weaken the quality of the findings. Furthermore, both groups were compared in terms of serum indicators, adverse events, OS and PFS, and overall clinical effect in this study, other indicators such as quality of life of the patient’s post-operation, 3- and 5-year survival rates could be further investigated.

## CONCLUSION

For patients with unresectable HCC, TACE combined with lenvatinib results in a high clinical efficacy with reduced AFP and VEGF levels, higher two years survival rate, and acceptable incidence of adverse events.

### Authors’ contributions:

**JL** conceived and designed the study.

**LL, XY, XL and LQ** collected the data and performed the analysis.

**JL** was involved in the writing of the manuscript and is responsible for the integrity of the study.

All authors have read and approved the final manuscript.
